# Management of Field-Evolved Resistance to Bt Maize in Argentina: A Multi-Institutional Approach

**DOI:** 10.3389/fbioe.2018.00067

**Published:** 2018-05-25

**Authors:** Ana M. Signorini, Gustavo Abratti, Damián Grimi, Marcos Machado, Florencia F. Bunge, Betiana Parody, Laura Ramos, Pablo Cortese, Facundo Vesprini, Agustina Whelan, Mónica P. Araujo, Mariano Podworny, Alejandro Cadile, María F. Malacarne

**Affiliations:** ^1^Dow AgroSciences Argentina S.R.L, Buenos Aires, Argentina; ^2^Dupont-Pioneer Argentina, Buenos Aires, Argentina; ^3^Monsanto Argentina S.R.L, Buenos Aires, Argentina; ^4^Grower Adviser (Member of Consorcio Regional de Experimentación Agrícola -CREA Brochero), San Luis, Argentina; ^5^Servicio Nacional de Sanidad y Calidad Agroalimentaria, Rosario, Argentina; ^6^Dirección de Biotecnología-Ministerio de Agroindustria, Buenos Aires, Argentina; ^7^Instituto Nacional de Semillas (INASE), Buenos Aires, Argentina; ^8^Asociación Semilleros Argentinos, Buenos Aires, Argentina

**Keywords:** Bt-maize, field evolved resistance, *Diatraea saccharalis*, sugarcane corn borer, Argentina, mitigation plan, insect resistance management (IRM)

## Abstract

Evolution of resistance to control measures in insect populations is a natural process, and management practices are intended to delay or mitigate resistance when it occurs. During the 2012/13 season the first reports of unexpected damage by *Diatraea saccharalis* on some Bt maize hybrids occurred in the northeast of San Luis province, Argentina. The affected Bt technologies were Herculex I® (HX-TC1507) and VT3PRO® (MON 89034 × MON 88017^*^). Event TC1507 expresses Cry1F and event MON 89034 expresses Cry1A.105 and Cry2Ab2, whichr are all Bt proteins with activity against the lepidopterans *D. saccharalis* and *Spodoptera frugiperda* (MON 88017 expresses the protein Cry3Bb1 for control of coleopteran insects and the enzyme CP4EPSPS for glyphosate tolerance). The affected area is an isolated region surrounded by sierra systems to the northeast and west, with a hot semi-arid climate, long frost-free period, warm winters, hot dry summers, and woody shrubs as native flora. To manage and mitigate the development of resistance, joint actions were taken by the industry, growers and Governmental Agencies. Hybrids expressing Vip3A protein (event MIR162) and/or Cry1Ab protein (events MON 810 and Bt11) as single or stacked events are used in early plantings to control the first generations of *D. saccharalis*, and in later plantings date's technologies with good control of *S. frugiperda*. A commitment was made to plant the refuge, and pest damage is monitored. As a result, maize production in the area is sustainable and profitable with yields above the average.

## Introduction

Insect pests are one of the main causes of losses in agriculture. Many different tools are available for farmers to manage insect pests. Since 1998 in Argentina maize farmers have grown transgenic hybrids that express insecticidal proteins derived from *Bacillus thuringiensis* (Bt maize) as an effective and environmentally friendly tool for integrated pest management (IPM). Evolution of resistance to control measures in insect populations is a natural process, and insect resistance management (IRM) practices are intended to delay or mitigate resistance when it occurs. One of the key measures for delaying evolution of resistance is the implementation of a refuge area in a Bt plot. The refuge is a portion of the field planted with non-Bt seeds where susceptible insects can survive to preserve susceptible alleles in the population. In the case of Bt maize in Argentina, the recommended refuge proportion is 10%. During the 2012/13 season the first case of field evolved resistance to Bt maize occurred in Argentina. *Diatraea saccharalis* (sugarcane borer, SCB) produced greater than expected damage on two Bt technologies: Herculex I® (Hx-TC1507) and VT3PRO® (MON 89034 × MON 88017) (Signorini et al., 2017; Grimi et al., 2018), both events express Bt proteins with activity against SCB and *Spodoptera frugiperda* (fall armyworm, FAW). The area affected is an isolated region and was not part of the major maize producing area until 2005 when farmers invested in pivot irrigation together with a high rate of Bt technology adoption.

When the unexpected damage was detected in 2013 farmers contacted technology providers to understand the situation and obtain recommendations on mitigating yield loss. The companies (Dow AgroSciences, DuPont Pioneer and Monsanto) worked together through the Argentina Seed Associations with the farmers and with the multiple governmental agencies involved in the approval and commercialization of transgenic crops (also known as genetically modified organisms GMOs) in Argentina. This article describes the joint actions taken by the industry, growers and Governmental Agencies to manage and mitigate this first case of resistance to a Bt crop in Argentina and the resulting improvement in management of SCB.

## Description of the problem situation

### Environment and agro-ecologic description

The department of Ayacucho in the northeast of San Luis province, where the greatest damage was detected, is geographically isolated by mountains from other maize producing areas. It has a dry environment, low rainfall with high temperatures in summer and mild winters with long frost-free periods and natural shrubby vegetation. Crop management under these environmental conditions is atypical of maize production areas in Argentina. Maize production requires irrigation and planting occurs during an extended period from September to January, with double cropping possible. These practices result in maize crops that are available almost year-round and more attractive to insects than other vegetation in the region. The agro-ecological characteristics of the area and the installation of pivot irrigation made this area a good place for production of maize seed making this a profitable activity that occupied between 30 and 50% of the area planted with maize.

### Bt adoption and technology management

**Figure 1:**
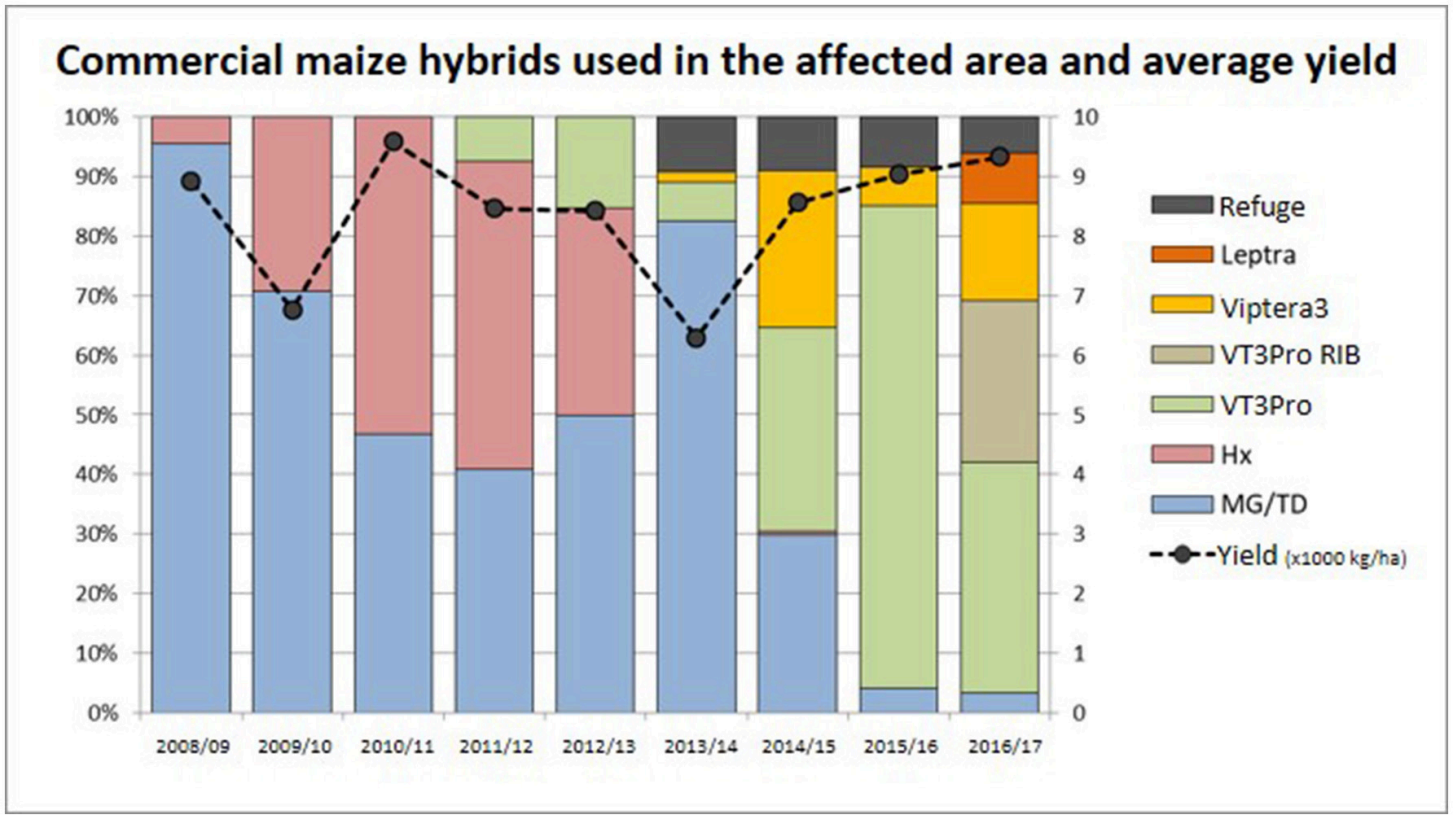
Technologies planted and average yield for maize in each season in the affected area. The bars represent the percentage of each technology planted in the affected area, and the dotted line indicates the average yield obtained for maize in the same area in thousand kilograms per hectare (Source: A. Cadile, CREA Brochero). [Leptra is the marketing name for the breeding stack of maize events TC1507 × MON810 × MIR162 × NK603. Viptera3 here refers to the breeding stack of maize events Bt11 × GA21 × MIR162. MG/TD here refers to Cry1Ab-Bt maize corresponding to events MON810 (MG) or Bt11 (TD)].

These environmental conditions and agricultural practices led to intense insect pest pressure in northeastern San Luis. This high insect pressure limited the planting date of maize, only to September and beginning of October, timing that has a high demand for irrigation. Upon commercialization, Bt maize provided a new tool for growers to manage lepidopteran pests that was more effective and simpler to deploy than insecticidal sprays and cultural practices. Accordingly, adoption of Bt technologies in the area was very rapid, especially for late-season plantings in which FAW is the major pest. By allowing late planting dates, a greater efficiency in the use of irrigation water was achieved, and yields were stabilized. Herculex I was launched in 2005 and provided very good control of SCB and FAW and farmers adopted this technology extensively across all the planting dates. In 2010 VT3PRO was commercially approved and adoption was again rapid (Figure 1).

In most cases, IRM practices (e.g., refuge adoption, crop rotation, weed management, insect monitoring, and insecticide applications when pest populations reached economic thresholds) were not emphasized with growers. These factors are similar to other resistance cases reported in the world and may have contributed to an unusually high selection pressure for SCB populations to evolve resistance northeastern San Luis.

### Damage

Upon reports of unexpected damage in HX or VT3PRO hybrids by growers, the technology providers visited the affected area to identify the damage, identify the pest species involved, and ensure that seed quality was not an issue. Once the pest was identified as SCB and the hybrids were confirmed to be HX or VT3Pro several fields were scouted to understand the extent (number of hectares) and the severity of the damage. Results showed that the affected area included nearly 11,000 hectares, including 9,000 ha with pivot irrigation in intensive agriculture and 2,000 ha of lower technology and land irrigation.

**Figure 2:**
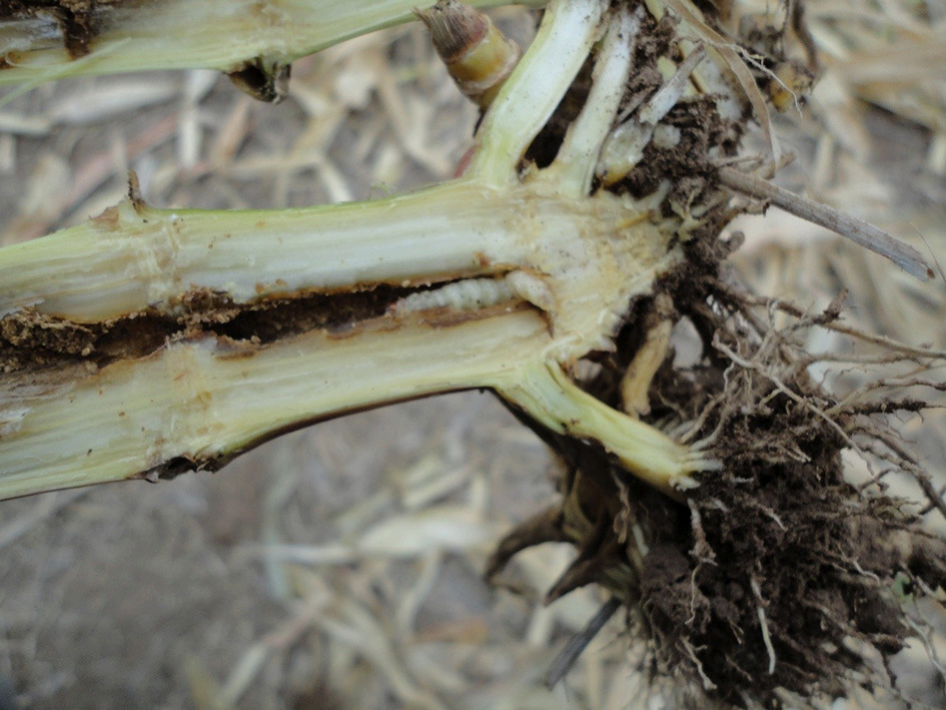
Damage caused by *Diatraea saccharalis*.

The damage found consisted of severely bored stems, tunnels that were over one meter long, and often more than one gallery per stem. This caused plant stem breakage, poor grain filling (low grain quality), and reduced yield (Figure 2). Sugarcane borer damage in stubble from the previous year indicated that the situation had existed for at least one season.

Insects surviving on maize containing these events are called “resistant biotype” after confirming the resistant phenotype (Signorini et al., 2017; Grimi et al., 2018) since no populations genetics studies have been done to determine whether all the insects in the whole geographic area consists of a single genetic population.

## Parties involved

As with any case of reduced benefits of a technology, many parties are affected and involved in different ways. In the case of field-evolved resistance of a target pest to a Bt crop, the main parties affected are farmers with resistant populations in their fields, technology developers, and, in Argentina, governmental agencies that regulate different aspects of the agricultural productions. For field-evolved resistance in a main target pest to Bt maize, three governmental institutions are related with different involvement: the Directorate of Biotechnology, the National Seed Institute, and the Directorate of Surveillance and Monitoring.

The Directorate of Biotechnology (*Dirección de Biotecnología*, DB)^1^ (within the scope of the Secretary of Food and Bioeconomy within the Ministry of Agroindustry) is in charge of the environmental risk assessment for the release of a GMO into the agricultural environment upon consultation with the National Advisory Commission on Agricultural Biotechnology (CONABIA). The CONABIA is constituted by representatives of the public and private sector involved in agricultural biotechnology. It is an interdisciplinary and inter-institutional group. It is recognized that resistance of target insects to Bt crops is not a biosafety risk but rather causes a reduction in the value or benefit of the Bt crop. Accordingly, the environmental assessment of an event for commercialization includes a section on IRM as an institutional way of helping preserve the benefits that the Bt technologies have for the environment and agricultural production in Argentina (Resolución SAGyP N° 701/11)^2^.

The National Seed Institute (*Instituto Nacional de Semillas*, INASE) oversees the proper execution of the Seed Law in Argentina. Among other objectives, the INASE issues resolutions related to seed production, hybrid registration, certification of seeds from origin, and packaging and labeling of the final product (seed bags). INASE is a member institution of CONABIA, thus it is involved also with GMO regulations.

The Directorate of Surveillance and Monitoring (*Dirección de Vigilancia y Monitoreo*, DVyM) within the National Service for Agri-food Health and Quality (*Servicio Nacional de Sanidad y Calidad Agroalimentaria*, SENASA) is in charge of the Argentine National System of Pest Monitoring and Surveillance (SINAVIMO), whose general goal is to provide updated and trustful information on the status of plant and animal pests, including those with impact on the productivity of the crops and cattle. Additionally, at the time of the sugarcane borer resistance case (2012/13), this Directorate received also the technical support of the National Commission of Resistant Pests (*Comisión Nacional de Plagas Resistentes*, CONAPRE).

The Argentine Seed Association (ASA) is a chamber that joins more than 80 seed companies, developers and licensees of agrobiotechnology, in the country. ASA coordinates member discussions on common themes that affect seed industry, like regulatory affairs, intellectual property and stewardship topics, among others. It has a technical and communication working group on IRM that is integrated by member companies with the main goal of preserving Bt technologies.

Most of the growers in the affected area, members of CREA Brochero, were grouped in an “Irrigation Group” that had regular meetings where they exchange experiences and knowledge. The existence of this group facilitated the alignment between growers and the work with the industry and governmental agencies.

## Approaching the problem together

Since many of the stakeholders described above have common interests in the general scope of agriculture and productivity for Argentina, there is a long history and experience of all parties working together. Field evolved resistance of sugarcane borer to Bt maize in San Luis was a new situation for which a solution had to be reached with a multi-institutional partnership.

After the detection and confirmation of unexpected damage to Bt maize by SCB, several meetings between growers, industry and governmental agencies took place over the following months. The situation was communicated to the Maize Chain Association and Growers Associations in August 2013, and academic experts were consulted to gather as many perspectives as possible for this first case in Argentina. The existing relationship between ASA and governmental agencies, which started in 1998 when the first Bt maize was commercialized in Argentina, was key for this multi-stakeholder collaboration to be effective.

Initially, the technology providers and growers were trying to assign blame to one another, and were reluctant to identify their own mistakes. Over the course of these meetings, a common understanding emerged that the situation had to be approached together by all the parties involved as agricultural community with a common goal: sustain maize productivity in the affected area (as maize is a key component in the system) and as far as possible keep the resistance confined to the original location.

A mitigation plan was discussed based on the previous characterization, and on the risk of spread of the resistant biotype. Several measures were proposed, some of them very radical and difficult to implement except in specific cases (not planting maize, tillage, not planting wheat or other SCB hosts, etc.). Other proposed measures were easier to implement. A plan for monitoring pest damage was developed with thresholds set for the application of chemical insecticides. For this purpose, a service company was hired to monitor around 9,000 ha included in the plan. The monitoring included, at least, a weekly visit to each field to detect egg masses, and upon detection of 10% plants with colored egg postures (indicating less than a week to hatching) persistent products were recommended for chemical control according to well established threshold by referent entomologists (Iannone and Leiva, 2015). A commitment was made to plant the refuge, and it was decided that Bt materials expressing different modes of action (e.g., Cry1Ab or Vip3A protein as in event MIR162) would be used exclusively (as they had not been affected by the resistant biotype). During the first season of the plan (season 2013/14), there was high FAW pressure, which caused severe damage to Cry1Ab maize, even in early plantings (since Cry1Ab maize does not provide protection from high FAW populations). Maize productivity of that season was the lowest in the last 10 years. Due to this situation, the following season it was agreed to use Cry1Ab or Vip3A technologies (single or stacks) in early planting to control the first generations of SCB, and in late planting it was necessary to use events with good control against FAW. Thus, technologies containing TC1507 or MON89034 events, either as single or stacks (such as Leptra or VT3Pro), had to be reintroduced in the affected area and new events as Viptera3 (containing Vip3A and Cry1Ab Bt proteins) were introduced to control this relevant pest (Figure 1).

This Mitigation Plan was communicated by ASA to the three governmental agencies. The DB together with CONABIA collaborated by providing technical assistance coming from their experts, and by helping delineate new regulations for IRM for Bt crops. The development of the Guideline (Circular N°7/2014) was coordinated by the DB and the actors involved in carrying this task were CONABIA, the National Institute of Agricultural Technology (INTA), SENASA, INASE and ASA (representing the Bt seed providers from the private sector). This multiparty collaboration was essential to determine the information necessary for Bt developers to provide.

The DVyM also took an active role as the Institution with expertise in the field of pest resistance. Their previous experience in cases of weed resistance to herbicides (especially glyphosate-resistant *Sorghum halepense* in Argentina) was of great guidance for the development of the Mitigation Plan and the coordination of the multi-institutional work.

INASE took a proactive role in the confinement of the resistant biotype to the affected area. As the Agency responsible for the certification of seeds from origin, it issued a Resolution to prevent seed production in the affected area with the aim of avoiding the escape of larvae inside the cobs toward the regions where the seeds are conditioned and bagged (mostly in the maize belt of Argentina). The first Resolution (N° 328/2013)^3^ was issued as early as September 2013, and annually renewed until its latest update in 2016 that made the measure permanent until new information becomes available that could justify the release of the restriction. This is a key measure from the containment perspective, but it produced many complaints by farmers that used to plant maize for seed production in the area (30–50% of maize area before the resistance outbreak).

Additionally, INASE involved its Summer Crops Commission (CONASE) for the understanding and support of additional IRM measures, leading to a Resolution to enable the seed blend refuge strategy in Argentina (Resolution N° 112/2014)^4^. The seed blend refuge, also known as refuge in the bag (RIB), is an alternative strategy of refuge where the Bt and non-Bt seeds are already mixed in the bag so that when farmers plant the bag they are planting Bt and its refuge at the same time. For the current Bt maize commercialized in RIB format in Argentina, the blend refuge is a mixture of 10% non-Bt seeds and 90% Bt seeds. This refuge strategy makes the operative part easier for the farmer while it guarantees the refuge will be more effective since it is planted at the same time as the Bt plants, with hybrids of similar maturity and agronomic practices and in the right proportion. So far, refuge seed blends are recommended for the areas in Argentina where SCB is the main pest since interplant movement by larvae is limited.

## Current situation

Despite the presence of sugarcane borer resistant biotype, at present damage caused by sugarcane borer and fall armyworm is limited and maize production in the area is sustainable and profitable with yields above the average. IPM and refuge education and training are continuing, together with INASE's prohibition of maize seed production in the affected area. Currently maize can be planted from the beginning of September to January and the Bt technology used is selected considering the planting date, the position in crop rotation, and neighbor crops. Damage by both SCB and FAW, is limited. The refuge adoption is high: 75% of the area planted with Bt maize included the corresponding refuge in 2013/14 season, increasing to 87% of compliance in 2014/15 season. Figure 1 reflects this successful compliance showing that 10% of the volume planted with maize corresponded to refuge in season 2013/14 to 2015/16 and the addition of RIB for VT3Pro in season 2016/17 (data provided by CREA Brochero, Figure 1). Except for some refuge management, it is practically not necessary to make insecticide applications to manage these pests, when the technology is well positioned in the system.

## Conclusions

This case generated many learnings that need to be emphasized. From the agronomical standpoint, the first learning showed that all parties involved had been working on the simplification of the agronomic practices, while the system is indeed complex. With this simplification of the system, the selection of resistance was accelerated, leading to a rapid loss of benefits. From a social perspective, another learning is that in order to preserve new technologies education is a key factor, and for Bt technologies this includes training farmers on Best Management Practices and improving refuge compliance. Preservation of the technology can only be reached through collaboration among all parties involved.

Resistance management and mitigation preserves the benefits of Bt crop technologies. This experience shows that with appropriate management practices maize can still be produced sustainably in an area where resistance to Bt events has occurred. The mitigation plan implemented by farmers, industry and government has been successful in limiting the spread of the resistant biotype. This positive scenario can only be reached because all parties involved have joined efforts toward this common goal.

## Author contributions

All authors listed have made a substantial, direct and intellectual contribution to the work, and approved it for publication.

### Conflict of interest statement

The authors declare that the research was conducted in the absence of any commercial or financial relationships that could be construed as a potential conflict of interest.

## Footnotes

^1^Dirección de Biotecnología (2014) Circular N°7. Directrices para la evaluación de los ítems D13 y D14 del formulario de segunda fase.

^2^Ministerio de Agroindustria (2011) *Resolución SAGyP N*° *701/11* Available online at: https://www.agroindustria.gob.ar/sitio/areas/biotecnologia/solicitudes/___experimental/_archivos/resolucion%20OVGM%20701-2011.pdf.

^3^INASE (2013). *Resolución N*° *328/2013*: Available online at: http://servicios.infoleg.gob.ar/infolegInternet/anexos/215000-219999/219950/norma.htm.

^4^INASE (2014). *Resolución 112/14:* Available online at: http://servicios.infoleg.gob.ar/infolegInternet/verNorma.do?id=230560.
